# Diagnosing and Preventing Q Fever in Regional New South Wales, Australia—A Qualitative Exploration of Perspectives of General Practitioners

**DOI:** 10.1111/ajr.70030

**Published:** 2025-03-14

**Authors:** Sachith Maduranga, Lok Bahadur Shrestha, Braulio Mark Valencia, Graeme Horton, Michelle Guppy, Heather F. Gidding, Stephen Graves, John Stenos, William Rawlinson, Andrew R. Lloyd, Chaturaka Rodrigo

**Affiliations:** ^1^ School of Biomedical Sciences UNSW Sydney Sydney New South Wales Australia; ^2^ Kirby Institute UNSW Sydney Sydney New South Wales Australia; ^3^ School of Medicine and Public Health University of Newcastle New South Wales Australia; ^4^ School of Rural Medicine University of New England Armidale New South Wales Australia; ^5^ School of Population Health UNSW Sydney Sydney New South Wales Australia; ^6^ Australian Rickettsial Reference Laboratory Geelong Victoria Australia

**Keywords:** Australia, general practitioners, New South Wales, Q fever, qualitative

## Abstract

**Objective:**

To qualitatively explore the perceptions of general practitioners in regional New South Wales, Australia, on diagnosing, managing and preventing Q fever.

**Setting:**

Q fever is a prevalent zoonosis in regional New South Wales, but diagnosis may be missed as patients have symptoms similar to influenza or COVID. Perspectives of general practitioners who are the primary health care providers in rural areas are important to understand the logistical difficulties in providing optimum care to Q fever patients.

**Participants:**

General practitioners practicing outside of metropolitan Sydney in regional postcodes of New South Wales, Australia.

**Methods:**

Eligible general practitioners were interviewed online using a semi‐structured interview guide on their approach to diagnosis, management and prevention of Q fever. The data were transcribed, coded using NVivo software, and analysed to identify emerging overarching themes.

**Results:**

Thematic saturation was achieved after 11 interviews. Diagnostic delays due to prioritising more common differential diagnoses for an influenza‐like illness, difficulties in navigating the complex serological test interpretations for diagnosis, logistical difficulties in arranging immunisation, and the need for continuing medical education were the broad themes emerging from the data analysis.

**Conclusions:**

Investment in continuing medical education and expansion of the reference resources made available to general practitioners regarding the diagnosis and management of Q fever will improve health care for people suffering from and at risk of Q fever in regional New South Wales.

**Summary**

- What this paper adds
  ○ This work for the first time qualitatively explores perspectives of General Practitioners on diagnosing, managing and preventing Q fever.
  ○ Diagnostic delays were very likely unless patients presented with clear risk factors, and complexity in interpreting serological tests added to the diagnostic problems.
  ○ Removing barriers to vaccination and continuous medical education programs was suggested as ways to reduce Q fever incidence and diagnostic delays in regional New South Wales.
- What is already known on this subject
  ○ Q fever is a highly infectious zoonosis common in regional Australia that is often missed due to symptoms' similarity with more common infections like influenza and COVID.
  ○ There is no published research exploring the perspectives of general practitioners regarding the barriers to diagnosing and preventing Q fever.
  ○ Perspectives of General Practitioners are important because they are the first‐contact health care workers in rural communities.

## 1 Introduction

Q fever is a highly infectious zoonosis typically presenting as an influenza‐like illness (ILI) in uncomplicated symptomatic disease [1, 2]. However, most infections are asymptomatic [3]. The disease is caused by the intracellular bacterium *Coxiella burnetii* , with a large reservoir in both domesticated animals (cattle, sheep, goats) as well as native animals (such as kangaroos and wallabies). People who are exposed to infected animals during work or recreational activities (e.g., farmers, abattoir workers, hunters and veterinarians) are at an increased risk of infection [2, 4]. Given that the pathogen is highly infectious (a single inhaled organism may cause disease) but is largely asymptomatic in infected animals, outbreaks associated with the arrival of an infected herd or flock are common [4]. Unfortunately, as there are many more common differential diagnoses for an ILI (e.g., influenza, COVID), Q fever may not be considered in the first presentation to a primary healthcare worker, even in areas with relatively high prevalence of Q fever and among high‐risk occupations.

Consequences of missed Q fever are significant both at individual and community levels. Most individuals with acute Q fever will recover within 2–6 weeks without long‐term sequelae, though a few may have serious organ involvement (e.g., pericarditis and meningoencephalitis). Less than 5% of patients progress to chronic Q fever, where endocarditis with associated valvular heart disease and osteomyelitis are known complications [5, 6]. Additionally, 10%–15% of patients may have chronic fatigue syndrome following acute infection [7]. At the community level, undetected or misdiagnosed infections can eventually cause large outbreaks, as once seen in the Netherlands between 2007 and 2010, when > 4000 cases were reported [8]. Though Q fever has been reported in almost every country in the world, the data on the actual burden of disease is sparse, and only Australia has approved a vaccine for use in humans. Therefore, it is a largely neglected topic in infectious diseases, which may translate to poor awareness among the primary healthcare workers who make first contact with ill patients in the community. Once correctly diagnosed, the infection can be easily treated by inexpensive and readily available antibiotics like doxycycline [9]. Thus, removing barriers to timely diagnosis of Q fever will lead to direct and indirect healthcare benefits for at‐risk communities.

We previously conducted a seroprevalence study of missed Q fever by testing residual plasma/serum from microbiologically undiagnosed patients with an ILI, living in regional New South Wales in Australia to determine the fraction of missed diagnoses in patients presenting to primary care [10]. However, after testing 542 eligible serum samples referred to NSW Health Pathology Randwick campus from undiagnosed patients between 2016 and 2023, only one missed acute Q fever case was confirmed. During the same period, from the same demographic, 731 samples were referred to the same diagnostic service for Q fever testing, and of these, 70 were positive for acute Q fever. Thus, in this sample, the clinical judgement of general practitioners was considered generally reliable as to when to request Q fever testing. However, there were two main limitations to these observations. Firstly, it is unknown what proportion of patients with suggestive symptoms for Q fever were referred for testing; and secondly, most samples tested for missed Q fever did not necessarily originate from known hotspots for the disease in regional NSW (and hence the missed cases may be underestimated). Thus, this qualitative study sought to investigate how general practitioners (GP) approach a potential Q fever diagnosis to further identify enablers and barriers to making this diagnosis in regional NSW. The interviews explored their knowledge, attitudes and practices in diagnosing, managing and preventing Q fever.

## 2 Methods

### 2.1 Pre‐Interview Consultations and Drafting the Interview Guide

Consultations were held with academic general practitioners (authors MG and GH) with expertise in general practice in regional NSW prior to developing the interview guide. The final draft of the interview guide had open‐ended questions covering the topics of diagnosis, management and prevention of Q fever. The aim of this semi‐structured interview guide was to allow the interviewees to freely express their ideas around pre‐selected topics, while ensuring consistency in structure across each interview session.

### 2.2 Interviews With General Practitioners

Invitations were sent to 200 publicly available email addresses of GP practices in regional NSW (outside greater Sydney postcodes) with 50 each from Southern (Shoalhaven and Illawara, Bega Valley, Eurobodalla, Southeast and Tablelands), Northern (New England and Northwest, North Coast), Central Coast and Hunter, and Western (remainder of NSW not mentioned above) regions of NSW. This email briefly introduced the researchers and the purpose of the study to potential interviewees, and those responding to this invitation email were sent a detailed explanation of the study in the form of a Participant Information Sheet and a Consent form approved by a Human Research Ethics Committee. All interviews were conducted online via Zoom, and the raw audio files were retained (for auto‐transcript). Only the interviewer and the interviewee were present during the interview, which lasted between 30 and 40 min. Subsequently, the transcription was corrected against the audio file by the interviewer. Each general practitioner was only interviewed once. Authors CR (MD, PhD), SM (MBBS), LBS (MBBS, MD) and BMV (MBBS, PhD) conducted the interviews. All interviewers were medically trained (although not general practitioners) males whose clinical experiences may have influenced the interpretations. CR was a researcher‐academic trained in internal medicine, BMV was a post‐doctoral scientist also trained as an infectious diseases' physician, and SM and LBS were PhD students at the time of conducting the interviews. For calibration, all authors participated in a pilot interview led by CR. There was no sample size calculation, and the interviews were conducted on a first come first served basis until thematic saturation was achieved.

### 2.3 Data Analysis

The data were analysed according to Braun and Clarke's (2006) 6 phases of thematic analysis [11]. After familiarising themselves with the data as mentioned above, initial coding and searching for themes was done by CR and SM independently; review and finalisation of themes were done by CR, LS, BMV and SM in consultation with AL. All authors contributed to the final report. The coding was done using NVivo (version 14, Denver, CO, USA). The reporting in this manuscript follows the Consolidated criteria for reporting qualitative research (COREQ) [12].

## 3 Results

**FIGURE 1:**
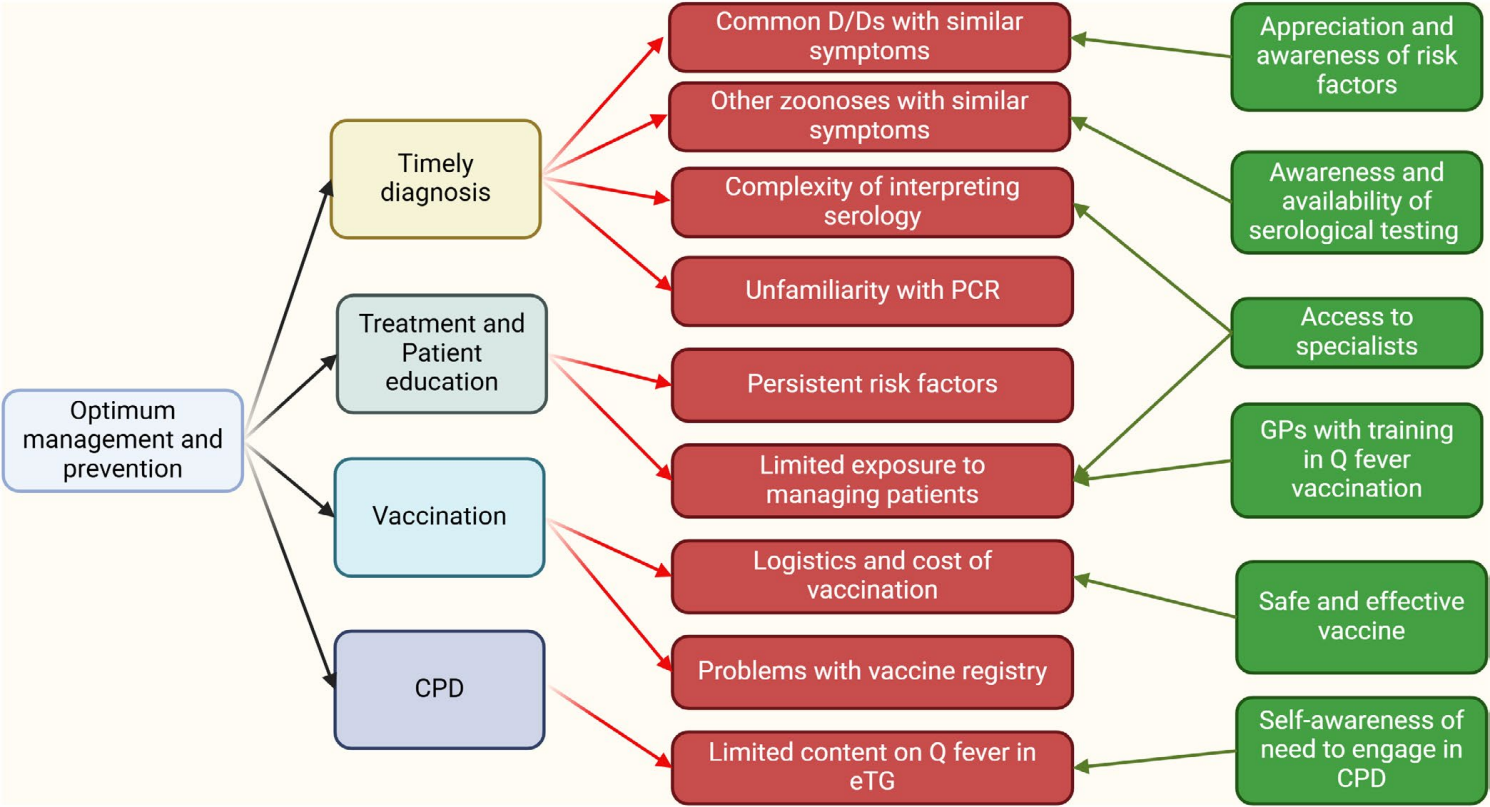
A tree diagram of information categories that emerged from the data coding of the interview transcript. Four main categories were further divided into subcategories of enablers (in green) and barriers (in red). The five themes emerging from the overall analysis (not shown in figure) are discussed in the text under results.

Eleven interviews (4 males, 7 females) were conducted between April and August 2024 with general practitioners (n: 10) or GP trainees (n: 1). The duration of their careers as medical doctors since graduation varied from 4 to 35 years (Table 1). They had been in general practice (or in training) from 1 to 30 years. Four participants had completed their undergraduate degrees overseas while the remainder graduated in Australia. Seven participants were within the 20–40 age brackets while all others were in the 41–60‐year‐old bracket. Four participants were from the Northern regional postcodes while three were from the Western regional postcodes. Two participants each were from Southern and Central Coast postcodes. The frequency of acute Q fever cases diagnosed or suspected by these interviewees in the last 12 months varied from zero to five. The data categories that emerged after the analysis of coded information are summarised in Figure 1. After interrogating these data categories, the following themes emerged.

**TABLE 1:** Demographic characteristics of interviewees.

Interviewee number	Age (years)	Sex	Postcode	Medical practice	GP	Training
01	41–60	Male	Redacted	35 years	30 years	Local
02	20–40	Male	Redacted	10 years	1 year	Overseas
03	20–40	Female	Redacted	7 years	< 1 year	Overseas
04	20–40	Female	Redacted	6 years	2 years	Overseas
05	20–40	Female	Redacted	9 years	5 years	Local
06	41–60	Female	Redacted	7 years	2 years	Local
07	20–40	Male	Redacted	9 years	4 years	Local
08	20–40	Female	Redacted	4 years	2 years	Local
09	20–40	Male	Redacted		2 years	Overseas
10	41–60	Female	Redacted	35 years	18 years	Local
11	41–60	Female	Redacted	30 years	25 years	Local

### 3.1 Enablers and Barriers to Suspecting Q Fever in a Patient With an ILI

All interviewees agreed that Q fever would not be among the first three most likely diagnoses initially considered in a patient who presents with an ILI. Common cold, COVID, influenza, parainfluenza, and respiratory syncytial virus (RSV) infections were cited as the top differential diagnoses considered (Q1—Q4, Q: Reference quotes in Table S1), while none mentioned a bacterial pathogen. Diagnosing a bacterial infection was more context specific depending on the age and findings of the history and examination (Q5, Q8) or if the patient did not get better by the second (Q8) or sometimes by the third visit (Q11). This sentiment was based on what is most observed in their practice and that patients with an ILI often get better on symptomatic treatment alone (Q6, Q7). Thus, it appears unlikely that a diagnosis of Q fever would be considered in the first visit unless a specific risk factor was identified (Q5, Q8) despite all interviewees acknowledging that Q fever patients can present without any pre‐existing risks.

The interviewees also reported that if specific epidemiological or occupational risks (farmers, veterinarians, zoo and abattoir workers, hunters) were identified, or if the patient was still unwell after the initial visit or sometimes multiple visits, then Q fever may be suspected, but it is just one of many infections that would be tested for in such a patient. Brucellosis, Leptospirosis, Toxoplasmosis, atypical pneumonias, and arboviral infections including Japanese encephalitis virus and cytomegalovirus infections were also considered as likely differential diagnoses by some of the interviewees in that scenario (Q8, Q9, Q10, Q11).

### 3.2 Enablers and Barriers for Confirming Diagnosis of Q Fever

Once suspected, all interviewees mentioned Q fever serology would be tested to make a diagnosis though none was comfortable in interpreting the test results without additional help. With one exception, none spontaneously mentioned Phase1 and 2 antibody responses. Also, none of the interviewees mentioned how acute and chronic Q fever cases can be differentiated by serology. There was very limited spontaneous discussion on different antibody subclasses with only one interviewee referring to IgM and a four‐fold rise in IgG antibodies (Q12), while none talked about IgA antibodies. Most interviewees admitted they would only request testing as “Q fever serology” without further specifications (Q13, Q14).

Once the results returned, interpretation would typically require some help, but all GPs interviewed named a reliable source that they can immediately refer to for additional help in confirming a diagnosis. The sources of help cited included the guidance provided on the test report itself and, when that was unavailable or unhelpful, they consulted the electronic therapeutic guidelines (eTG), an infectious diseases specialist (Q15, Q18) or a microbiologist as needed. Of all the resources named, eTG was the most popular (Q16, Q17, Q18, Q19).

Requesting a PCR test to diagnose Q fever was, however, not as popular, with only one interviewee having ever requested one for Q fever (Q21). Most interviewees were not familiar with the role of PCR in diagnosis (Q22) except for the suggestion that a negative PCR for other common respiratory pathogens would trigger consideration of Q fever, or thought that the laboratory would do a PCR if serology was suggestive of Q fever (Q20). None of the interviewees were also familiar with the seroconversion time window within which a PCR test may be positive.

### 3.3 Treatment and Patient Education

All interviewees were familiar with doxycycline as the preferred first line treatment for Q fever. None of the interviewees had treated special groups such as pregnant women or immunocompromised patients. Four interviewees who were among the most experienced GPs interviewed had encountered people with confirmed or suspected chronic Q fever who required specialist care. Even then the number of patients diagnosed was only a handful with two people reporting one diagnosis each and others using descriptors like “I've had a couple…”, and “we have got a few people”.

As for patient education, the commonly raised topics of discussion were the risk of complications of chronic Q fever, avoiding Q fever vaccine after a confirmed infection, compliance with the course of prescribed antibiotics (and information on drug side effects), warning of post‐infective fatigue, and reassurance that it can be cured (Q23, Q24, Q25). Some acknowledged that it is difficult to advise on prevention because the risk was closely linked to their occupation or residence, which is difficult to change (Q23).

### 3.4 Vaccination: Enablers and Barriers

All interviewees were aware of the Q fever vaccine and that it could not be given without an antigen challenge test, although only five interviewees had completed the additional training and/or had enough experience to do the vaccinations themselves. The consensus was that it is a safe and efficacious vaccine (Q26, Q29, Q31) with only one adverse event reported by the five who had done vaccinations (Q26). Those who had done vaccinations could recall the procedure in detail (performing a sensitivity test, and then vaccination on a second visit) and explained the potential barriers for vaccination, including the out‐of‐pocket costs and the limited number of practices offering vaccinations. They also welcomed the recent inclusion of the Q fever vaccine into the Australian Immunisation Registry (AIR) because it reduced paperwork and improved patient safety by avoiding the risk of double vaccination (Q32, Q33). Those who had not done vaccinations were unaware of these finer details (Q27, Q28, Q30). Not all GPs in a practice or not all practices within a postcode were performing Q fever vaccinations. This task seemed to be delegated to “trained/experienced” practices or GPs, probably due to logistical issues of time and costs of performing sensitivity testing (Q27, Q29). One interviewee recalled the historical government‐funded Q fever vaccination drive (now discontinued) as a great initiative which improved vaccination rates among farmers (Q30).

### 3.5 Need for GP Education on Q Fever

The need for Q fever content to be incorporated into Continuous Medical Education (CME) activities was universally accepted by all interviewees. However, the opinions on priority for Q fever in CME activities, duration and frequency of such activities, mode of delivery and proposed content differed (Q35, Q36, Q37, Q38). The GPs trained in Australia thought that local training makes them more familiar with Q fever compared to their colleagues trained overseas, but even Australian GPs who have moved to rural practice from a metropolitan area felt that they were unfamiliar with Q fever (Q36, Q37). Some GPs opined that more frequently encountered conditions merit priority in CPD activities (Q39) and that refreshers on Q fever should primarily be targeted at those practicing in regional areas. There were also misconceptions that people without a Medicare card cannot have their Q fever vaccinations recorded in AIR (Q34). Activities limited to an hour or two per year on the topics of signs and symptoms, diagnostic testing, vaccination, and complications were suggested by the interviewees, although each person had different ideas as to which topic was more important (Q37, Q38, Q40). Most preferred online activities, although some felt face‐to‐face activities would allow better engagement and networking. Most GPs referred to the eTG as their go‐to source on Q fever content but felt it was inadequate and needed to be expanded (Q38). Awarding CPD points was highlighted as a method to encourage participation.

## 4 Discussion

This qualitative analysis of general practitioner experiences in diagnosing, managing and preventing Q fever in regional New South Wales, Australia, identified several themes emerging from the data analysis: Q fever is unlikely to be diagnosed on the first visit of an ILI presentation in the absence of risk factors; positive diagnoses are not encountered by general practitioners frequently; almost all diagnoses are based on serology, for which external help is often needed in interpreting test results; the Q fever vaccine is perceived as safe and effective, but there are barriers to its uptake; there is a need for Q fever to be included in onboarding and continuous professional development programs for GPs, at least for those practicing in regional areas.

In a typical ILI presentation, GPs appropriately prioritised common viral infections as differential diagnosis. As some interviewees acknowledged it is not unreasonable to adopt a “watch and wait” approach as most such infections are self‐limiting with the caveat that in the elderly or immunocompromised acute influenza or COVID should be diagnosed by RAT or PCR testing and treated with antiviral drugs. Given the difficulty in obtaining a GP appointment a proportion of ILI patients may not even attend a GP practice. Therefore, unless strong epidemiological and occupational risk factors suggest it, testing for Q fever in the first GP visit is unlikely even in regional NSW, where GPs are familiar with the illness. Thus, it is likely that the apparently “low” rate of diagnosed Q fever cases reported by all interviewees may be an underestimate of the true prevalence due to a combination of the above factors. This has implications for diagnostic tests because a *C. burnettii* PCR test is likely to become negative within 2 weeks after the onset of symptoms [13], and therefore the GPs nominated reliance on serology is appropriate within the context of their practice.

The Q fever vaccine was perceived by interviewees as effective and with limited side effects, but problems were noted in the logistics of service delivery and record keeping. According to those interviewed, not all GPs were experienced in doing vaccinations, presumably related to the time‐consuming nature of the pre‐testing, the jurisdictional requirement of additional training, and the need for multiple appointments to complete the vaccination process [14, 15]. From the GPs' standpoint, this “specialisation” leads to less waste and cost to practice (and patients), but it may also limit accessibility to those wanting to get vaccinated. It also meant that the GPs who did not do vaccinations were unfamiliar with details of the process and hence would have limited capacity to advise interested patients. The cost of vaccination to patients was considered high when serology, skin testing, and multiple appointments were included. For those who require vaccination due to employment, these costs may be covered by their employer, but otherwise it is a likely a deterrent that prevents high vaccine uptake. The inclusion of the Q fever vaccine in AIR was appreciated by all interviewees doing Q fever vaccinations, but one person (incorrectly) noted that it cannot be used for people without a Medicare card. Thus, clear communication of how this information can be recorded for those without a Medicare card (using an individual health identifier linked to My Health Record) may be worth adding to existing resources such as the training module on Q fever diagnosis and vaccination available via the Australian College of Remote and Rural Medicine (worth 2 CPD points) [16].

Q fever is a neglected but a widely prevalent infection globally [5, 6, 17, 18, 19, 20, 21] which is probably underdiagnosed due to a lack of awareness both among patients and medical practitioners [22]. Interestingly, although an effective vaccine is available, Australia is the only country that has approved it for use [23]. Compared to other pathogens that cause an ILI such as COVID, influenza, and RSV, the volume of research on Q fever is limited. It was not possible to find prior qualitative studies that evaluated the perceptions of primary healthcare workers including general practitioners on diagnosing, managing and preventing Q fever to understand the real‐life difficulties they face in reducing the Q fever burden. The very few qualitative studies available from literature were only focussed on patients and at‐risk occupational groups like farmers or husbandry workers [24, 25]. The only qualitative study that interviewed healthcare workers from Australia included a range of professionals in occupational health, environmental health, and specialists in infectious diseases, but not primary healthcare workers who play a critical role in diagnosing cases in the community [26]. In the public health system in Australia (and in many other countries with similar systems) GPs are the first point of contact for patients and specialists can only be accessed via their referrals (unless in private healthcare or after a hospital admission). Hence, this study addresses a critical knowledge gap in informing how Q fever detection and prevention can be further improved in grassroots community healthcare settings.

This study had several limitations. Qualitative research is context specific, and this work is based on the interviews of GPs from regional NSW, Australia. The context, facilities‐related issues, and other logistical issues faced by GPs in other countries or other states of Australia may be different. While this impacts on the generalisability of the findings, it still provides a baseline framework and data that can be compared against for future studies on the same topic. It is likely that the GPs who responded to our request for interviews were those who had a special interest in Q fever, and they may not be representative of all GPs in regional NSW. However, the issues highlighted by them are likely to be relevant to all GPs in regional NSW. The prevalence of Q fever is not homogenous across regional NSW, and the GPs interviewed did not always practice in a Q fever hotspot. Those practicing in high‐prevalence settings may be more aware of the illness and diagnose it quickly. Hence, in this study, we asked about the number of cases diagnosed in the last 12 months as a proxy measure of familiarity with the infection, and the numbers were low for all interviewees regardless of the postcode of practice. Finally, as this is a qualitative study, it is not possible to draw any conclusions on the prevalence of Q fever or vaccine uptake from this data.

The findings of this study have implications for clinical practice and future research. In terms of practice, there is an opportunity to engage general practitioners from rural NSW in CPD activities or reintroduce or expand (e.g., eTG) the existing training materials linked to CPD points. Eventually, this may lead to more GPs undertaking vaccinations as they become more aware of the illness and confident in vaccinating. In research, it would be interesting to see if the same findings are true for general practitioners in other states of Australia because the jurisdictional laws, local training opportunities and disease prevalence would be different between the states. Furthermore, qualitative interviews engaging wider representation of the community, including other community and hospital‐based healthcare workers, patients, members of the community, and policymakers, will paint a more wholesome picture of enablers and barriers to prevent and manage Q fever in rural Australia.

## 5 Conclusion

This qualitative exploration of GP experiences in diagnosing, managing and preventing Q fever in regional NSW identified several important themes, including the difficulty of diagnosing Q fever in the first visit as more prevalent differential diagnoses must be prioritised, the need for additional support in interpreting Q fever serology, logistical and record‐keeping problems with regard to vaccinations, and the acceptance of the need for continuous professional development activities on the topic. This work fills a critical knowledge gap in improving services to Q fever patients and general practitioners in regional NSW and sets the bar for future qualitative studies of a similar nature in Australia and globally.

## Author Contributions

**Sachith Maduranga:** investigation, data curation, formal analysis. **Lok Bahadur Shrestha:** investigation, data curation, formal analysis. **Braulio Mark Valencia:** investigation, formal analysis, data curation. **Graeme Horton:** methodology, writing – review and editing, resources, supervision. **Michelle Guppy:** methodology, writing – review and editing, supervision, resources. **Heather F. Gidding:** conceptualization, methodology, writing – review and editing. **Stephen Graves:** conceptualization, writing – review and editing, supervision. **John Stenos:** conceptualization, supervision, writing – review and editing. **William Rawlinson:** conceptualization, writing – review and editing, supervision. **Andrew R. Lloyd:** conceptualization, supervision, resources, methodology, funding acquisition, writing – review and editing. **Chaturaka Rodrigo:** conceptualization, investigation, funding acquisition, writing – original draft, methodology, formal analysis, project administration, data curation.

## Ethics Statement

This research was approved by the Human Research Ethics Committee of the Southeastern Sydney Local Health District (2021/ETH10995), and all participants provided informed written consent.

## Conflicts of Interest

Andrew Lloyd is a member of, and Heather Gidding has previously contributed to the Seqirus advisory board. The authors declare no conflicts of interest.

## Supporting information


Appendix S1.

## Data Availability

The data that supports the findings of this study are available in the Supporting Information of this article.
