# Supplementary material for: Diagnosing and Preventing Q Fever in Regional New South Wales, Australia—A Qualitative Exploration of Perspectives of General Practitioners
Source: Aust J Rural Health. 2025 Mar 14;33(2):e70030. doi: 10.1111/ajr.70030 (PMC11907686; doi:10.1111/ajr.70030)
Supplement: Supplementary file 1 — Appendix S1. [file AJR-33-0-s001.docx]

### Supplementary Table 1. Quotes from the transcripts of general practitioner interviews referenced in the results

| **Quote number** | **Interviewee number**  **(Table 1)** | **Quote** |
| --- | --- | --- |
| Suspecting Q fever | | |
| Q1 | 06 | *“So, for an influenza like illness I’d consider COVID, Influenza, adenovirus, other coronaviruses, parainfluenza”* |
| Q2 | 08 | *“At the moment my pick would be influenza, COVID and RSV”* |
| Q3 | 11 | *“Obviously, influenza, COVID and then probably parainfluenza”* |
| Q4 | 02 | *“Obviously influenza, RSV and then of course COVID”* |
| Q5 | 01 | “*You know, if they're farmers or things like that, that could be one of those type of infections. So probably if I thought that was a presentation, I would be doing serology Obviously, you want to take an occupational history people. See if they're from that sort of type of background. So, a lot of people living in this area do have potential for exposure. So, your index of suspicion might be a little bit higher than it would be otherwise….I think it can be quite hard to differentiate acute fever from other acute viral illnesses*.” |
| Q6 | 02 | “*If we feel that they* (patients) *are going to improve within next few days, no red flags in the history, examination, we don't do anything, just, you know, symptomatic management*” |
| Q7 | 05 | “*If it's a viral illness, and they're not that unwell, I don't order any (investigations), because it doesn't change my management. Depending on their job, so if they work in healthcare or with disabled people or children, I may order a nasal swab or PCR* (for respiratory viral infections)” |
| Q8 | 11 | “[*if someone then didn't settle down within the few days that you would expect an influenza, illness or a covid illness to settle. Then you'd definitely be looking for other things.*](https://unsw-my.sharepoint.com/personal/z3488606_ad_unsw_edu_au/Documents/Transcribed%20Files/Interview%2004.m4a)*... if they were doing high risk* [*activities like pigging*](https://unsw-my.sharepoint.com/personal/z3488606_ad_unsw_edu_au/Documents/Transcribed%20Files/Interview%2004.m4a) *or* (Kanga-) *roo shooting, a*[*nd particularly if they're butchering the animals at the time, then*](https://unsw-my.sharepoint.com/personal/z3488606_ad_unsw_edu_au/Documents/Transcribed%20Files/Interview%2004.m4a) [*they're going to be high risk for Q fever*](https://unsw-my.sharepoint.com/personal/z3488606_ad_unsw_edu_au/Documents/Transcribed%20Files/Interview%2004.m4a)*… it's persisting a bit longer. It doesn't settle down in 3-4 days.* (If) *They're getting worse, you know, instead of better you're thinking Q fever, or Lepto* (-spirosis) *and brucellosis cause they're popular”* |
| Q9 | 10 | “… *I'd be thinking about things like, ..CMV. Toxo* (-plasmosis)*. Sort of atypical but significant presentations… where people are more unwell*.” |
| Q10 | 05 | “*If I think there have enough risk factors, I will draw blood for Q fever, leptospirosis, brucellosis, arbovirus serology and JE* (Japanese Encephalitis) *virus*” |
| Q11 | 07 | “…*and by then (second visit) I will be doing the atypical pneumonia tests as well which includes pertussis so second visit will probably prompt more investigations, and probably a third visit before I start thinking about zoonotic diseases*.” |
| Confirming a diagnosis of Q fever | | |
| Q12 | 02 | “*We generally do the Q fever serology that is IgM and IgG. Then we do get the results within… few days*” |
| Q13 | 05 | “*I generally just ask for a Q fever serology, which takes like a week to come back*” |
| Q14 | 09 | “*I'm not familiar with the specifics of testing, but in terms of a blood test I can go with like Q Fever serology. I understand that there's Q fever culture (*of *C. burnetii) in theory, but I believe that is something that a restricted kind of laboratory would do*.” |
| Q15 | 01 | “*It's quite complicated. I would seek help and seek advice, maybe from an infectious disease’s registrar…If I get positive cases, I often get a call from public health*.” |
| Q16 | 03 | “*It's mostly to the eTG* (electronic therapeutic guidelines) *that I refer to*” |
| Q17 | 04 | “*We have our guidelines on the eTG website. So that normally has I think all the information regarding treatment .... We can still contact, for example, the hospital to get advice on treatment. and they normally do contact tracing”* |
| Q18 | 05 | “*eTG is what I usually refer to I would just ring ID* (infectious Diseases Department) *which is at John Hunter (hospital) and discuss it with them*” |
| Q19 | 06 | “*I look at eTG and we have a very good ID specialist who is more than happy to chat*” |
| Q20 | 02 | “*If it's positive… the lab, does it sometimes, yeah,* (if) *the IgM is positive, I think they generally do test for PCR*” |
| Q21 | 05 | “*I have* (requested a Q fever PCR)*, but I do sometimes forget that I can* (request it).” |
| Q22 | 10 | “*I believe you can do a PCR. I don't know. Honestly, I've never ordered a PCR before for Q fever*” |
| Treatment and patient education | | |
| Q23 | 04 | “…*just to look out for symptoms, seek help early on if there's recurrent symptoms. And then yeah, the difficult thing is most people that are exposed to Q fever, it’s because where they live or what they're doing, and it's not something you can easily change. But that's something we normally discuss, like how can you minimise the risk*” |
| Q24 | 05 | “*I give them advice regarding the antibiotic like, take it with food, and that it can cause sensitivity to sun, heartburn and reflux. I tell them to get a work certificate so that they can rest at home and hydrate*.” |
| Q25 | 10 | “*I'd probably follow them up fairly regularly for a while to make sure that they, you know, responded to the antibiotics and that their symptoms were improving. I think they need to know that there is the possibility of long-term problems. Be that cardiac, chronic fatigue or whatever. That would probably be covered in the information sheet I would give them*.” |
| Vaccination | | |
| Q26 | 01 | “*My personal experience with* (It is that its a) *good vaccine, you know, very low incidence of significant side effects, I think, in all of my time giving it here, which has been 5 years, we might have had one*.” |
| Q27 | 02 | “*Not me, but in our practice, there are couple of GPs who does* (vaccinations)*, but not by myself. I know they do a skin test before giving the vaccine. And after that, depending on the result, they decide whether the patient needs the vaccine*” |
| Q28 | 04 | “*No, I know there is a vaccine. I know it's has not been around really long. I think relatively new vaccine, but no, I wouldn't know about how* (to administer it)*.*” |
| Q29 | 05 | "*I'm accredited to do them. I've done the testing where you get the patients, take the blood and do the skin prick antigen test and get them back in 7 days to read the skin test and serology, and then, if both are negative, then I give the Q fever vaccine. It seems that younger people are a bit more open to getting it and anyone that goes hunting, they will tend to get it. you need to bunch patients together because in order to make it worth it, because you have to buy a vial of the antigen. So, you must buy that, and it wouldn't be worth me just vaccinating one person for Q fever. So, you basically must bunch up, you must get, maybe at least 5 patients in order to make it worth it. And then all those 5 patients must be able to come back at the same time. It's all very tedious. You must make sure you get it (mixing and diluting) right,…and it all takes a lot of time. Then the patients must pay out of pocket for the vaccine, which cost about $120, so the cost can be a bit prohibitive. The patients (also) must pay for the appointment. The initial one to come and get skin prick test done and for the blood test, and then they must come back again and pay for getting vaccinated. So, at the end of the day its about $300 out of pocket for the whole thing*." |
| Q30 | 10 | “*Some years ago, there was a publicly funded vaccination program for Q fever, which is a very good thing. It ran for a couple of years and I think a lot of farmers were vaccinated then*” |
| Q31 | 11 | "*There's a couple of different groups that are coming to get the Q fever vaccination. We've got lots of people that are coming from overseas that have been brought to the area by the abattoir to work in the abattoir, and so most of them have not had Q Fever, but you find it more in the people that have lived locally for a long time and worked on properties. The effectiveness seems quite high, I can't say that I've ever seen someone with Q fever that's been previously vaccinated. That's not my experience. So, it must be effective. Because we got a big abattoir with hundreds of people working at it. And I've never seen anyone with Q fever* (from there)." |
| Q32 | 01 | “*I've had huge problems with the* (Previous) *register system. That like, vaccine - good, registers - terrible. But the registration process is stopping as of next week, which is great. It might be interesting to see what happens now as the paperwork is reducing*” |
| Q33 | 05 | “*It's dangerous because it's a once in a lifetime vaccine. So, you could have people that inadvertently would get another one, because a lot of patients sometimes don't remember. So, I think it's really good. I was really pleased to see that now it's incorporated into AIR*.” |
| Q34 | 11 | “*I think that's a good idea. We've had some difficulty with that, because a lot of the people come from overseas to work at the abattoir. They don't have a Medicare number. We need it to upload to AIR. So that's difficult. We're trying to work out how we do that. We can put it onto our own computer system, and we can give them piece of paper that says, yes, you've been vaccinated to give to work. But it's not necessarily going through to AIR because they don't have an individual health identifier*.” |
| Q35 | 06 | “*If I were to go to a CPD session, I probably would want to know about vaccination. I think it's one of those conditions which we all fear, but often forget. And so further education is important*.” |
| Q36 | 02 | “*Q fever was a like a new thing for me, because I used to work in urban and metropolitan areas. We hardly saw any Q fever cases* (There)… *I think it's a really good opportunity to improve your knowledge… about the vaccines and complications. Given that, it's not a really common presentation, I would say. at least once a year would be a good option*.” |
| Q37 | 03 | “*In my region. I think it is fairly important, because there's a large farming community. Probably not as relevant as frequently seen non-communicable conditions, for example. But then, yeah, it's definitely higher up on the scale compared to an urban area*” |
| Q38 | 08 | “*I guess the main question is, should we be testing more for Q fever? And I guess just a bit of update on things like electronic like therapeutic guidelines, which is arguably our bible for GP's. I think there is very limited information* (on Q fever) *other than the fact that what antibiotic to use and for how long. I don't think there's any clear guideline on if as soon as patients fall into a risk group, do you test for it* (Q fever)? *or is there a clear indication list of when to test or not rather than or when to consider it*? " |
| Q39 | 01 | *“There's lots of modules which you could always look up. I suppose the questions really got time to do it…. think we're already saturated with educational opportunities”* |
| Q40 | 02 | *“Regards to Q fever given that, it's not a really common presentation, at least once a year would be a good option.”* |
